# Intervention and information effects at the individual level during the COVID-19 pandemic in Japan

**DOI:** 10.1371/journal.pone.0294189

**Published:** 2023-11-20

**Authors:** Mateus Silva Chang, Isamu Yamamoto

**Affiliations:** 1 Faculty of Economics, Keio University, Tokyo, Japan; 2 Faculty of Business and Commerce, Keio University, Tokyo, Japan; Universiti Malaysia Sarawak, MALAYSIA

## Abstract

This paper estimated the impact of intervention effects (state of emergency (SOE) or quasi-SOE requirements) and information effects (publicized increases in the number of coronavirus disease 2019 (COVID-19) deaths and fear of infection) on preventive behaviors and telecommuting during the COVID-19 pandemic using the Japan Household Panel Survey. Our results indicated that SOEs and quasi-SOEs had positive effects on the adoption of preventive behaviors among individuals, including handwashing, which indicates that an SOE has a direct effect and an indirect effect. Although SOEs in Japan were less enforceable and more lenient than those in other countries, they still had a certain effect on people’s adoption of preventive behaviors. However, the contribution of information effects was much larger than that of intervention effects, suggesting the importance of how and when information should be communicated to the public to prevent the spread of infection.

## Introduction

One of the government tools to slow the transmission of infectious diseases such as coronavirus disease 2019 (COVID-19) is the declaration of a state of emergency (SOE). However, according to previous studies [1–3] that examined the stay-at-home measure from daily smartphone location data aggregated at the county and prefecture levels, the effects of such interventions (“intervention effects”) are limited and smaller than those of voluntary behavioral changes based on information about the COVID-19 pandemic (“information effects”). This is the first study to use representative household panel data to investigate these two effects by examining individual-level behavioral changes. The household panel data enabled us to identify specific prevention behaviors, such as avoiding traveling, regularly washing hands, avoiding events and dining out, and working from home (telecommuting). Understanding the effectiveness of interventions and voluntary behavioral changes is extremely important to improve individuals’ responses. We studied the impact of SOEs and quasi-SOEs by controlling for individual attributes, number of COVID-19 deaths, and fear of infection.

## Data and methodology

We used samples from the initial four waves of the Japan Household Panel Survey COVID-19 special survey (JHPS-COVID19) and the Japan Household Panel Survey (JHPS) of 2021 in our empirical analysis. The JHPS is a representative Japanese household panel survey that is conducted every February. The main objective of the JHPS is to provide data that represent the Japanese population, allowing the analysis of dynamic behaviors by economic entities. The survey covers comprehensive topics such as household structure, individual attributes, academic background, employment status, time use, health conditions, well-being, income, wealth and others.

The JHPS-COVID19 was conducted in May and October in both 2020 and 2021. The objective of the JHPS-COVID19 was to collect representative information about the effects and perceptions of the COVID-19 pandemic on topics such as physical and mental health, time use, behaviors, economic status, and employment status.

Following the previous literature [2], we used the following equation:

$$y_{ijt}=\underset{Intervention\;effect}{\underset{︸}{\alpha_{1}D_{jt}(SOE)+\alpha_{2}D_{jt}(quasi‐SOE)}}+\underset{Information\;effect}{\underset{︸}{\beta_{1}m_{jt}+\beta_{2}{fear}_{ijt}}}+\gamma_{1}x_{ijt}+p_{j}+f_{i}+\varepsilon_{ijt},$$

where *i* is the individual, *j* is the prefecture of residence, and *t* is the time. The dependent variable *y*_*ijt*_ takes 1 if the respondent adopted a preventive measure. *D*_*jt*_(*SOE*) or *D*_*jt*_(*quasi*-*SOE*) is a dummy variable that takes a value of 1 when the SOE or quasi-SOE was adopted at least one day of the month in which the preventive behavior was reported or at least one day of the week in which telecommuting was reported. *m*_*jt*_ indicates the natural logarithm of the number of deaths due to COVID-19 in the month or week the dependent variable was considered. *fear*_*ijt*_ is a dummy variable that takes a value of 1 if the respondent had a fear of infection. As the JHPS-COVID19 contains information on the degree of fear, it was possible to directly examine the “fear effect” that was previously emphasized [2] as a form of the information effect. *x*_*ijt*_ represents individual attributes, *p*_*j*_ and *f*_*i*_ are prefecture and individual fixed effects, respectively, and *ε*_*ijt*_ is idiosyncratic disturbance.

### JHPS and JHPS-COVID19 data

**Fig 1:**
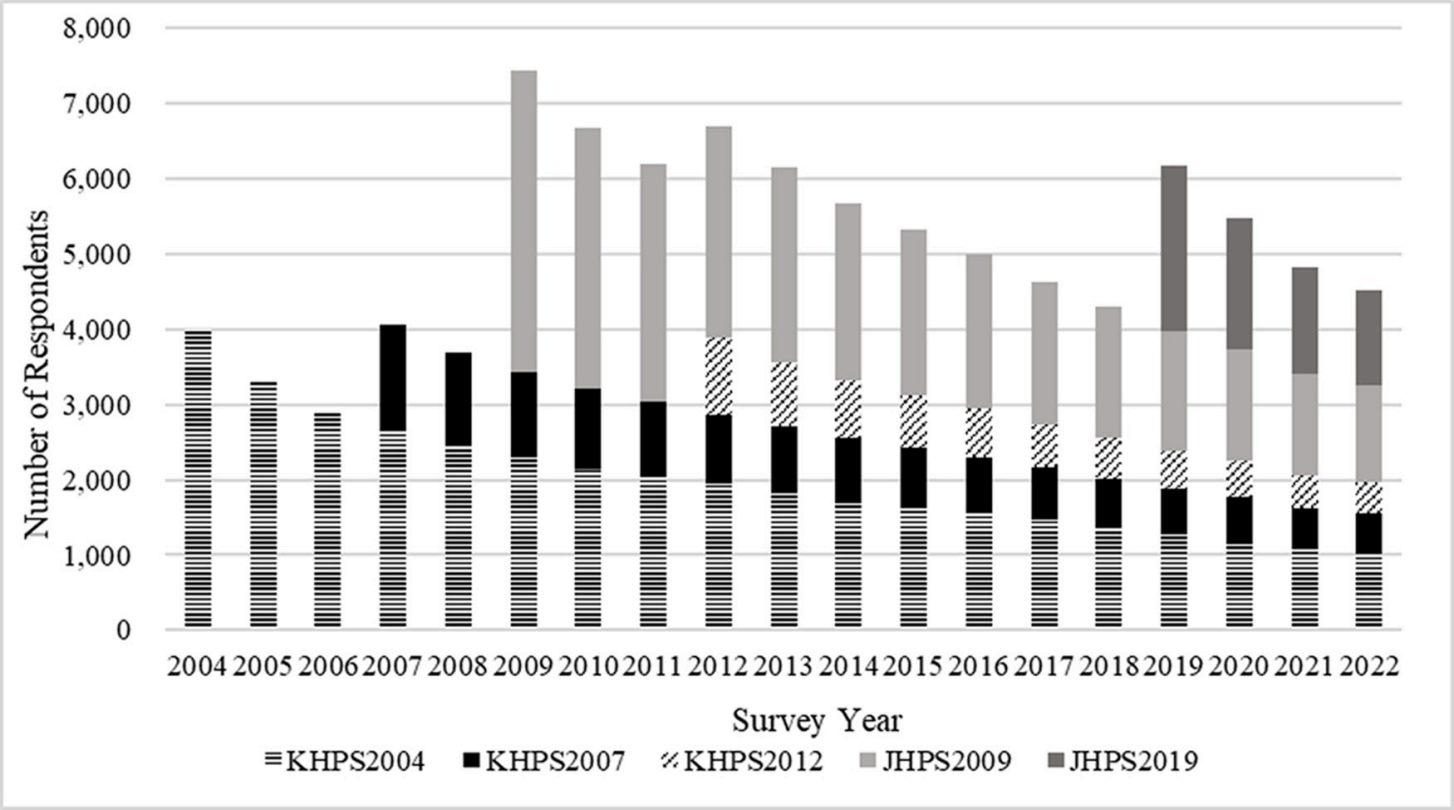
Sample size of the Japan Household Panel Survey. Source: [4].

The JHPS was established in 2014 as a result of the integration of the Keio Household Panel Survey (KHPS), a survey that has been implemented since 2004, and the former JHPS that was introduced in 2009. The first wave of the KHPS was conducted in 2004 and collected information from a sample of 4,005 respondents aged 20 to 69 years. To address the sample attrition problem, the KHPS sample was extended through the recruitment of an additional 1,419 individuals in 2007 and 1,012 individuals in 2012. The first wave of the former JHPS was conducted in 2009 and obtained data from 4,022 respondents aged 20 and over. Due to the similarity of these two surveys, the KHPS and the former JHPS were combined in 2014 and named the “Japan Household Panel Survey (JHPS)”, thereby indicating the adoption of a common questionnaire. After integration, the JHPS received a top-up sample of 2,203 respondents aged 20 and over in 2019. Fig 1 shows the total sample sizes of the JHPS for each survey year.

Aiming to provide data that represent the Japanese population, the survey subjects were selected according to a two-stage stratified random sampling method. Japan was stratified into 24 strata following a regional and municipal classification. The number of subjects in each stratum was allocated in proportion to the registered population according to the Basic Resident Register of the previous year. Next, the districts inside each stratum were selected following a systematic random sampling process, and an average of 10 subjects per district were randomly selected until the predefined total number of subjects per stratum was achieved. Reserve subjects were randomly selected (they had characteristics similar to those of the original subjects they were supposed to replace) and used, when necessary, to replace original subjects who could not be contacted or declined to participate in the survey [4].

Respondents were recruited in January and were informed about the anonymity and confidentiality of their answers. The participants received the questionnaires in their home (in February) after agreeing to join the survey [5]. Informed consent was not obtained, given that the data were analyzed anonymously. In addition, the survey was approved by the Institutional Review Board of the Institute for Economic Studies, Keio University (approval number: 22012R).

The JHPS-COVID19 was a special survey conducted with the respondents of the JHPS. The initial waves targeted the respondents from the JHPS in 2020, while subsequent waves targeted the respondents from the previous JHPS-COVID19. The exception was the third wave that targeted the respondents from the second wave and the respondents of the JHPS in 2021. Table 1 details the survey target, the number of total respondents, the response rates for each survey and the number of respondents in this study.

**Table 1:** Number of respondents and response rates for each survey.

Survey name	Survey date	Survey target (a)	Number of respondents in total (b)	Response rate (b)/(a)	Number of respondents in this study
**1**^**st**^ **JHPS-COVID19**	May/Jun 2020	Respondents of the JHPS2020 (5,470)	3,891	71.1%	3,799
**2**^**nd**^ **JHPS-COVID19**	Oct./Nov 2020	Respondents of the 1^st^ JHPS-COVID19 (3,891)	3,244	83.3%	3,198
**JHPS2021**	Feb 2021	Respondents of the JHPS2020 (5,470)	4,817	88.0%	4,815
**3**^**rd**^ **JHPS-COVID19**	May/Jun 2021	Respondents of the JHPS2021 plus 2^nd^ JHPS-COVID19 (4,988)	3,681	73.7%	3,678
**4**^**th**^ **JHPS-COVID19**	Oct./Nov 2021	Respondents of the 3^rd^ JHPS-COVID19 (3,681)	3,314	90.0%	3,306
**Total**	-	-	18,947	80.6%	18,796

Note: The number of respondents in total is the number of collected questionnaires that were completed by the respondents. The number of respondents in this study is the number of questionnaires after cleaning and merging the data.

### Study variables

***y***_***ijt***_ is a dependent variable and took a value of 1 if the respondent adopted preventive measures, including avoiding traveling, avoiding close and crowded spaces and close-contact settings (the 3Cs in Japan), regular handwashing, reducing contact with people by at least 70%, refraining from attending events and dining with people other than relatives, and teleworking. Respondents were asked whether they performed each prevention measure in April (JHPS-COVID19 first and third waves), September (JHPS-COVID19 second and fourth waves), or January (JHPS2021). They were also asked whether they worked from home for at least one day in the last week of the corresponding month.

***D***_***jt***_**(*SOE*) or *D***_***jt***_**(*quasi*-*SOE*)** is a dummy variable that took a value of 1 when the SOE or quasi-SOE was adopted at least one day of the month in which the preventive behavior was reported or at least one day of the week in which telecommuting was reported.

***m***_***jt***_ indicates the natural logarithm of the number of deaths due to COVID-19 in the month or week the dependent variable was considered. To account for the case of 0 deaths, the number of deaths plus 1 was logarithmically transformed. We confirmed that the use of an inverse hyperbolic sine transformation, as in previous studies [1–3], did not significantly change our results.

***fear***_***ijt***_ is a dummy variable that took a value of 1 if the respondent had a fear of infection. In each wave of the JHPS-COVID19 and JHPS2021, the participants were asked “How concerned are you about being infected?” and “How concerned are you about someone in your family being infected?” We determined that the respondents feared COVID-19 infection when the answer was “very concerned” or “somewhat concerned” for at least one of the questions.

***x***_***ijt***_ are the following individual attributes:

**Sex**: a dummy variable that took a value of 1 if the respondent was male.

**Marital status**: a dummy variable that took a value of 1 if the respondent was married.

**Age category**: respondents were separated into those aged 20 to 39 years, 40 to 59 years and those aged 60 years or older.

**Education status**: a dummy variable that took a value of 1 if the respondent completed tertiary education.

**Income category**: respondents were separated into quartiles according to their income level.

**Work status**: a dummy variable that took a value of 1 if the respondent worked.

**COVID-19 vaccination status**: a dummy variable that took a value of 1 if the respondent received at least two doses of the COVID-19 vaccine.

**High grit**: a dummy variable that took a value of 1 when the respondent’s Grit Scale score was above the average. The Grit Scale measures the extent to which individuals are able to maintain focus and interest and persevere in achieving long-term goals [6]. It is expected that a person with a high grit score would have a higher probability of adopting preventive behaviors since they would be more committed and capable of maintaining the adoption of behaviors to protect themselves and others.

### The nature of Japanese SOEs and quasi-SOEs

The Japanese government can declare an SOE and quasi-SOE at the prefecture level after considering three conditions: the infection situation (such as the number of newly reported cases), medical service system (such as hospital beds available), and surveillance system (such as PCR test situation). An SOE and quasi-SOE allow prefecture governments to take stock of emergency measures to curb the spread of COVID-19, such as refraining from unnecessary and nonurgent going out, reducing the business hours of restaurants and bars, and adopting telework to reduce the movement of the population. However, unlike countries such as Australia [7], the UK [8] and the US [9], a specificity of the Japanese SOE and quasi-SOE is the fact that only recommendations and not mandates can be adopted. A clear example of this difference is the fact that Japan did not adopt lockdown policies and recommended only that businesses close and the population stay at home. Interactive maps from the Oxford COVID-19 Government Response Tracker [10] illustrate this difference, revealing that during the pandemic, Japan was one of the few countries that adopted only recommendations.

Measures are more restrictive and stringent in an SOE than a quasi-SOE. For example, during an SOE, the government can recommend the reduction of business hours or business closures, while during a quasi-SOE, the former is allowed, but the latter is not possible [11, 12]. Another important difference is that SOEs are declared for a whole prefecture, but for quasi-SOEs, the prefectural governor can geographically limit this state to specific municipalities if necessary [11–13].

In February 2021, the government revised the law allowing the imposition of fines up to 300,000 yen for violators of reduced bar and restaurant business hours during the SOE and fines up to 200,000 yen during the quasi-SOE [11, 12]. However, there is no evidence that businesses were punished with such fines, despite reports of businesses that violated the law. Instead of relying on fines, the Japanese government adopted positive reinforcement by offering compliant businesses daily rewards of up to 200,000 yen.

According to the Japanese government definition, a quasi-SOE can be declared when the third stage (rapid increase in the number of infected people) in a four-stage classification of the prevalence of infection is achieved. If the number of infections continues to increase, advancing to the fourth and highest stage defined as an “explosive infection spread”, then the government is able to declare an SOE. Although decisions were made on a case-by-case basis, the government defined shortages of hospital beds (hospital bed occupancy rate of 20% and hospital admission rate of 40%) and weekly new infections (15 people per 100 thousand) as criteria to determine if a prefecture entered the third stage. The criteria to determine if a prefecture entered the fourth stage were also shortages of hospital beds (hospital bed occupancy rate of 50% and hospital admission rate of 25%) and weekly new infections (25 people per 100 thousand) [11].

### The evolution of the pandemic

**Fig 2:**
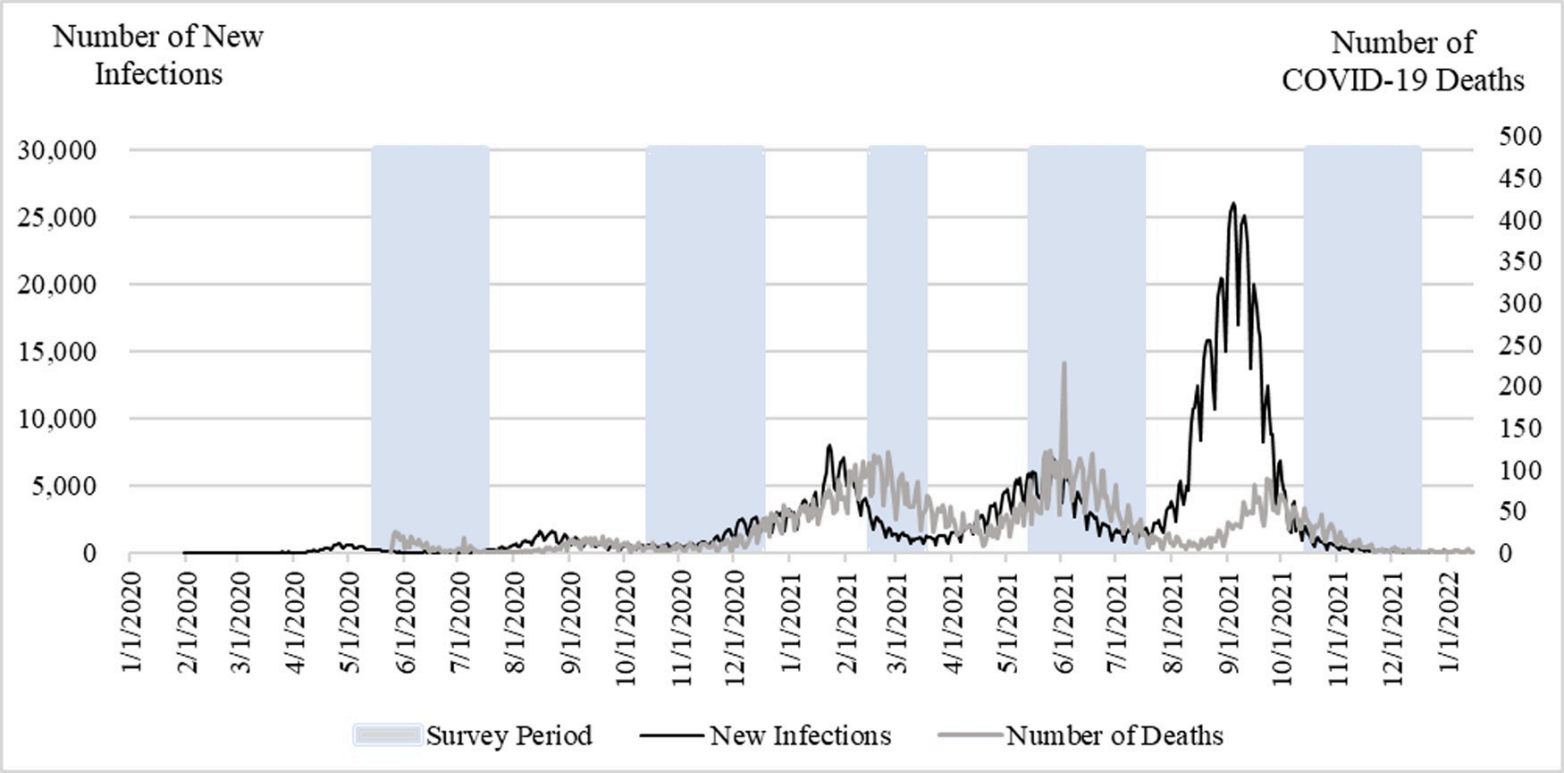
Number of new infections and deaths from COVID-19. Source: Author calculations based on [14].

Fig 2 illustrates the pandemic situation in Japan during 2020 and 2021. The number of new infections indicated that during the study period, there were five waves of COVID-19. The first cases of infection occurred in April 2020, and the highest peak of infections occurred in September 2021. The number of deaths from COVID-19 also indicated the existence of five waves, with the highest death rate occurring after the peak of the 4^th^ wave of infections. Coincidentally, the surveys were fielded in periods after infection peaks. The fourth wave was the exception, with the peak of infections occurring during the period the survey was fielded.

### Statistical analysis

We estimated the equation as a random-effects or fixed-effects linear probability model. If the coefficients *α*s (intervention effects) were significantly positive, intervention policies promoted the adoption of preventive behaviors. Similarly, if *β*s (information effects) were positive, exposure to COVID-19-related information (or the fear induced by the available information) encouraged people to adopt infection prevention actions. We analyzed the data using STATA/SE version 14.2 for Windows (StataCorp, College Station, TX, United States).

## Results and discussion

### Demographic characteristics and prevention measures

The urgency of understanding the effects of the unprecedented pandemic and the measures adopted by governments worldwide resulted in a myriad of studies focusing on topics ranging from socioeconomic and behavioral impacts to physical and mental health issues. One of the main issues of these studies, especially those related to prevention behaviors, is the availability of appropriate data to conduct the investigations, with many depending on cross-sectional data and rapid internet surveys with quota samples or convenience samples (people who did not have access to the internet were not able to participate in the survey). In other words, they depended on evidence based on correlations from cross-sectional data that did not necessarily represent the country population and lacked long-term behavioral follow-up [15]. Aiming to overcome these issues, we employed a large and high-quality longitudinal survey that, derived from probability samples, represented the Japanese adult population.

The characteristics of the sample are summarized in Table 2. The sex and age group distributions of the sample were similar to those of the Japanese population aged 21 years or older in 2020: 48.07% were males, and 51.93% were females; and 23.86% of participants were aged 20 to 39 years, 33.71% were aged 40 to 59 years, and 42.43% were aged 60 years or older according to the *2020 Population Census* (Statistical Bureau [16]). Despite the existence of missing values (approximately 2,100 cases), the sample’s average monthly household income was still representative (approximately 491 thousand yen), being slightly higher than the average of the Japanese population. According to the *Comprehensive Survey of Living Conditions* (Ministry of Health, Labour and Welfare), the average household income was 5,643 thousand yen in 2020 or approximately 470 thousand yen per month [17]. The share of respondents with a tertiary education level was slightly greater than that of the Japanese population, possibly given the existence of missing values for this variable (approximately 1,650 cases). According to the *2020 Population Census*, 41.7% of Japanese individuals with some level of education and aged 15 years or older had a tertiary education level [18]. In contrast, the share of respondents from Tokyo and the three neighboring prefectures (Chiba, Kanagawa, and Saitama) was also similar to that of the Japanese population aged 21 years or older, approximately 30% [16].

**Table 2:** Demographic characteristics of the respondents.

Variables	Group	N/mean	%/SD	Variables	Group	N/mean	%/SD
**Sex**	Men	8,703	46.3%	**SOE (telework)**	Yes	9,988	53.1%
	Women	10,093	53.7%		No	8,808	46.9%
**Age**	20s to 30s	3,160	16.81%	**Quasi-SOE (telework)**	Yes	1,194	6.35%
	40s to 50s	7,053	37.52%		No	17,602	93.65%
	60+	8,583	45.66%	**Log of the number of deaths**		3.161	1.717
	In years	56.59	15.78	**Fear of infection**	Yes	14,464	77.1%
**Marital status**	Married	13,323	71.8%		No	4,307	22.9%
	Single	5,228	28.2%	**Five preventive behaviors**	Yes	12,808	69.4%
**Education level**	Less than tertiary complete	8,765	51.1%		No	5,651	30.6%
	Tertiary complete	8,383	48.9%	**Avoiding traveling**	Yes	17,471	94.0%
**Household monthly income**	Q1	4,434	26.6%		No	1,121	6.0%
	Q2	4,092	24.5%	**Avoiding the 3Cs**	Yes	17,583	94.3%
	Q3	4,282	25.7%		No	1,058	5.7%
	Q4	3,864	23.2%	**Handwashing**	Yes	16,543	88.7%
	Total (ten thousand)	49.19	75.21		No	2,102	11.3%
**Worker**	Yes	12,291	68.1%	**Reducing contact with people**	Yes	14,988	80.6%
	No	5,757	31.9%		No	3,607	19.4%
**Two doses of a vaccine**	Yes	2,136	11.4%	**Avoiding events and dining out**	Yes	18,032	96.8%
	No	16,660	88.6%		No	594	3.2%
**High grit**	Yes	8,718	47.3%	**Telework**	Yes	1,918	17.5%
	No	9,730	52.7%		No	9,036	82.5%
**SOE (preventive behavior)**	Yes	10,041	53.4%	**Firm size**	Less than 500	7,846	68.1%
	No	8,755	46.6%		500+ or public agencies	3,679	31.9%
**Quasi-SOE (preventive behavior)**	Yes	1,303	6.9%	**Employment status**	Regular employee	5,714	58.8%
	No	17,493	93.1%		Nonregular employee	3,997	41.2%
**Region**	Tokyo plus three neighboring prefectures	5,641	30.0%				
	Others	13,155	70.0%				

Regarding prevention behaviors, in more than 90% of the observations, respondents avoided events and dining out, close and crowded spaces and close-contact settings, and traveling. Approximately 88% of the respondents regularly washed their hands, while 80% reduced their contact with people. The percentage of cases in which telework was adopted on specified dates was approximately 17.5%.

### Empirical results

The estimation results based on the random-effects model are summarized in Table 3. The dependent variable in columns (1)-(4) is a dummy that took a value of 1 if all five prevention measures were adopted, while it was a telework dummy in columns (5)-(8). Columns (1)-(4) show that both intervention effects (SOE and quasi-SOE) and information effects (number of deaths and fear of infection) were positive and statistically significant. As the addition of information effect variables resulted in only a small decrease in the coefficients of intervention effect variables, we focused on the full model in column (4). The implementation of an SOE increased the probability of adopting all five preventive measures by 6%, while the implementation of a quasi-SOE increased the probability by 7.1%, implying that the impact of an SOE and quasi-SOE was basically the same (the F test could not reject the null hypothesis that the coefficients of the SOE and quasi-SOE were equal), although a quasi-SOE imposes fewer restrictions on movement. In Japan, if a quasi-SOE was not enough to control the outbreak, an SOE declaration with stronger restrictions on movement was issued. Thus, it is possible to interpret that a quasi-SOE could have served as a threat of a stricter SOE and promoted people’s behavioral changes to take more prevention measures. This may imply that a phased intervention method with quasi-SOEs and SOEs as a policy response to the pandemic is effective.

**Table 3:** Estimation of intervention and information effects: Random-effects model.

	Five Preventive Behaviors				Telework			
	(1)	(2)	(3)	(4)	(5)	(6)	(7)	(8)
**Intervention Effect**								
SOE	0.074**	0.059**	0.075**	0.060**	0.076**	0.084**	0.076**	0.084**
	(0.008)	(0.008)	(0.008)	(0.008)	(0.008)	(0.008)	(0.008)	(0.008)
Quasi-SOE	0.079**	0.070**	0.080**	0.071**	-0.001	0.003	-0.000	0.003
	(0.014)	(0.014)	(0.014)	(0.014)	(0.013)	(0.013)	(0.013)	(0.013)
**Information Effect**								
Number of Deaths		0.036**		0.036**		-0.014**		-0.014**
		(0.003)		(0.003)		(0.004)		(0.004)
Fear of Infection			0.068**	0.064**			-0.003	-0.002
			(0.010)	(0.010)			(0.009)	(0.009)
**Individual Attributes**								
Male	-0.128**	-0.129**	-0.122**	-0.123**	0.084**	0.085**	0.084**	0.084**
	(0.011)	(0.011)	(0.011)	(0.011)	(0.011)	(0.011)	(0.011)	(0.011)
Married	0.059**	0.062**	0.052**	0.055**	0.007	0.006	0.007	0.006
	(0.013)	(0.013)	(0.013)	(0.013)	(0.013)	(0.013)	(0.013)	(0.013)
Age: 20s to 30s (base)								
Age: 40s to 50s	0.012	0.010	0.012	0.010	-0.016	-0.016	-0.016	-0.016
	(0.015)	(0.015)	(0.015)	(0.015)	(0.015)	(0.015)	(0.015)	(0.015)
Age: 60+	0.019	0.019	0.023	0.023	0.002	0.002	0.002	0.002
	(0.016)	(0.016)	(0.016)	(0.016)	(0.017)	(0.017)	(0.017)	(0.017)
Tertiary Education Level	-0.025*	-0.023*	-0.024*	-0.022*	0.107**	0.107**	0.107**	0.107**
	(0.011)	(0.011)	(0.011)	(0.011)	(0.011)	(0.011)	(0.011)	(0.011)
Income: Q2	0.000	-0.001	-0.000	-0.002	-0.001	0.001	-0.001	0.001
	(0.011)	(0.011)	(0.011)	(0.011)	(0.012)	(0.012)	(0.012)	(0.012)
Income: Q3	-0.002	-0.002	-0.002	-0.002	0.007	0.009	0.007	0.009
	(0.012)	(0.012)	(0.012)	(0.012)	(0.013)	(0.013)	(0.013)	(0.013)
Income: Q4	0.005	-0.000	0.005	-0.000	0.033*	0.037**	0.033*	0.037**
	(0.013)	(0.013)	(0.013)	(0.013)	(0.014)	(0.014)	(0.014)	(0.014)
Worker	-0.045**	-0.047**	-0.046**	-0.048**	0.010	0.023	0.012	0.025
	(0.011)	(0.011)	(0.011)	(0.011)	(0.051)	(0.051)	(0.052)	(0.052)
Two Doses of a Vaccine	0.006	-0.014	0.008	-0.012	-0.054**	-0.047**	-0.054**	-0.047**
	(0.010)	(0.010)	(0.010)	(0.010)	(0.009)	(0.009)	(0.009)	(0.009)
High Grit	0.034**	0.036**	0.036**	0.037**	0.033**	0.032**	0.033**	0.032**
	(0.011)	(0.011)	(0.011)	(0.011)	(0.012)	(0.012)	(0.012)	(0.012)
Sample Size	14,185	14,185	14,179	14,179	8,778	8,778	8,773	8,773
Number of Individuals	4,258	4,258	4,256	4,256	2,933	2,933	2,932	2,932

Robust standard errors in parentheses.

Statistical significance:

**p< 0.01 and

*p< 0.05.

Regarding the information effect, the estimated coefficients showed that a 1% increase in the number of deaths increased the percentage of people who adopted all five prevention behaviors by 0.036%. If we calculated the substitution rate of the SOE and deaths, the imposition of the SOE corresponded to an increase in the number of deaths by approximately 167% (6%÷0.036%) or approximately 1.7 times. Additionally, fear of infection led to an increase of 6.4% in the adoption of all preventive behaviors.

**Fig 3:**
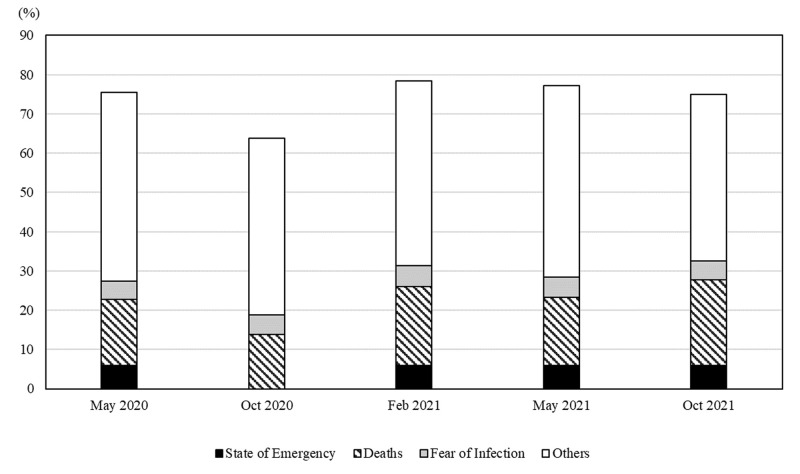
Decomposition of the contribution of intervention and information effects: The case of Tokyo. Note: The decomposition of the contribution of intervention and information effects was based on the estimations from column 4 of Table 3.

Following the previous literature [2], we decomposed the contributions of an SOE and quasi-SOE, number of deaths, fear, and other factors to the adoption rate of all preventive behaviors based on the coefficients in column (4). Fig 3 shows the decomposition results when we used each variable’s average values for Tokyo residents. The SOE contribution was approximately 5%, deaths 18%, fear 5%, and other reasons 46%, which indicates that the information effect was approximately 23%, while the intervention effect was approximately one-fifth (5%). If we suppose that other factors represented the contribution of usual or “new normal” prevention habits (even with no change in SOE status, deaths, or fear), they could be included in the information effect. In this case, the information effect totaled 69%, with the intervention effect becoming less than one tenth of the information effect. These results are similar to previous findings based on prefecture-level data [2, 3] in the sense that information effects have a greater impact on people’s behavior. To check for possible endogeneity bias in the model, we also used lagged data for the SOE, quasi-SOE, and number of deaths. The results were similar to the ones presented, so they were omitted for simplicity.

Given that the Delta variant had high transmissibility, we checked whether the spread of the Delta variant impacted the estimation results. As the Delta variant was first reported in Japan in March 2021 [19], we considered that the influence of the Delta variant was captured in the 3^rd^ and 4^th^ JHPS-COVID19 surveys. Therefore, as a robustness check exercise, we estimated the model without the data of the 3^rd^ and 4^th^ surveys (an estimation without the data of the 4^th^ survey, a period when the Delta variant was dominant, was also conducted). The estimation results were similar to those presented in Table 3, indicating no or very small influence of the Delta variant.

For other factors, the estimation result showed that people with the following characteristics tended to adopt preventive behaviors: female sex [20–24], married, 60 years or older [23, 24], people who did not work, and perseverant. In contrast, variables such as income and vaccination status were not statistically significant.

Next, columns (5)-(8) show that the declaration of an SOE increased the probability of adopting telework by 7.6 to 8.4%, while the coefficients of the quasi-SOE were not significant. These results indicate that a decision to conduct telework largely depended on an SOE declaration. Fear was not significant, while the number of deaths was negative and significant. Although it was difficult to interpret why the death coefficient was negative, its magnitude was extremely small (a 1% increase in the number of deaths results in a 0.014% decrease in the telework rate), being close to zero. Moreover, such small information effects are understandable if we suppose that a decision to implement telework depended more on companies than on individual workers.

Individual attributes indicated that the probability of implementing telework was significantly higher among males, individuals with a tertiary education level, high-income earners, and perseverant individuals, while vaccination status was significantly negative, showing that after the vaccination series was completed, there was a tendency to return to commuting.

Even in cases in which teleworking was implemented, its frequency could be different. To account for this, we conducted a robustness check analysis by replacing a telework dummy with the weekly number of days worked from home, which is available in each survey. We found no major changes from the estimation results in Table 3.

Furthermore, the availability of telework could change depending on the infection status and other factors. To account for this possibility, we controlled for the number of deaths due to COVID-19 in Table 3. It is also likely that the availability of telework increased as time passed after the outbreak of the pandemic because firms and workers would be able to prepare the equipment and network necessary for telework. To observe a difference by time, we conducted a robustness check analysis where we estimated the basic model without the 3^rd^ and 4^th^ survey data. We found no major changes from the original estimation results in Table 3.

Table 4 presents the estimation results for each preventive behavior based on the fixed-effects model. Columns (1) and (7) present similar results even after controlling for time-invariant region- and individual-specific characteristics. The exception is that fear coefficients were basically not significant. Coefficients from columns (2)-(6) indicate a difference between an SOE and quasi-SOE, with the latter being relatively smaller for avoiding traveling and not statistically significant for the 3Cs and avoiding events and dining with others. In contrast, the quasi-SOE coefficient for reducing contact with people was almost three times larger than that of the SOE, with the F test confirming that their difference was statistically significant. Thus, we can understand that this behavior’s contribution made significantly positive coefficients of the quasi-SOE in column (1) of Tables 3 and 4.

**Table 4:** Estimation of intervention and information effects: Fixed-effects model.

	Preventive Behaviors						Telework
	All	Each Preventive Behavior					
		Avoiding Traveling	Avoiding the 3Cs	Handwashing	Reducing Contact with People	Avoiding Events and Dining Out	
	(1)	(2)	(3)	(4)	(5)	(6)	(7)
**Intervention Effect**							
SOE	0.061**	0.048**	0.019**	0.013*	0.042**	0.017**	0.092**
	(0.008)	(0.005)	(0.004)	(0.005)	(0.008)	(0.003)	(0.009)
Quasi-SOE	0.067**	0.020*	-0.010	-0.017	0.113**	0.005	0.010
	(0.014)	(0.008)	(0.008)	(0.009)	(0.012)	(0.006)	(0.013)
**Information Effect**							
Number of Deaths	0.036**	0.002	0.007**	0.003	0.037**	0.004**	-0.012**
	(0.003)	(0.002)	(0.002)	(0.002)	(0.003)	(0.001)	(0.004)
Fear of Infection	0.004	0.015*	0.012	-0.003	0.018	0.009	0.003
	(0.012)	(0.007)	(0.007)	(0.008)	(0.011)	(0.005)	(0.011)
Sample Size	14,179	14,249	14,269	14,269	14,244	14,261	8,773
Number of Individuals	4,256	4,266	4,266	4,271	4,269	4,266	2,932

Robust standard errors in parentheses.

Statistical significance:

**p< 0.01 and

*p< 0.05.

Individual attribute variables were omitted for simplicity.

Interestingly, the SOE had a positive effect even on the likelihood of handwashing. The SOE had a direct effect on traveling, avoiding the 3Cs, reducing contact with people, and refraining from attending public events and dining out, such as prohibiting large-scale events and reducing restaurant and bar business hours. However, it was impossible for the government to directly intervene in the behavior of people’s handwashing. Thus, the positive effect of the SOE in column (4) should have reflected just the indirect announcement effect.

Regarding the information effect, an increase in the number of deaths increased the chances of avoiding the 3Cs, having contact with other people and joining events and dining out, while fearing infection increased the chances of avoiding travel.

We also investigated how the adoption of all preventive behaviors and telework in heterogeneous groups were affected by intervention and information effects. First, Table 5 indicates that the information effect was similar, while the intervention effect was noticeably larger for younger individuals, which is consistent with the previous literature [3]. From a geographical perspective, the intervention effect was positive and significantly larger in the region around the capital (Chiba, Kanagawa, Saitama, and Tokyo), where the population and number of infected people were larger, while the information effect was positive and significant only in the rest of the country. Regarding work status, the intervention effect of the SOE was greater for people who did not work, whereas the information effect of the number of deaths was greater for people who worked. Regarding firm size, the intervention effect was significantly greater for large firms and government agencies than for medium and small firms.

**Table 5:** Estimation of intervention and information effects by individual attributes: Fixed-effects model.

**(a) Preventive Behaviors**	Age Group		Region		Work Status		Firm Size	
	20–39	40+	Tokyo plus three neighboring prefectures	Rest of the country	Workers	Nonworkers	Less than 500	500+ or Public Agencies
	(1)	(2)	(3)	(4)	(5)	(6)	(7)	(8)
**Intervention Effect**								
State of Emergency	0.094**	0.055**	0.175**	0.042**	0.047**	0.076**	0.034*	0.059**
	(0.023)	(0.009)	(0.021)	(0.010)	(0.011)	(0.014)	(0.015)	(0.019)
Quasi-State of	0.110**	0.058**	0.135**	0.064**	0.071**	0.051*	0.045	0.102**
Emergency	(0.036)	(0.015)	(0.025)	(0.019)	(0.017)	(0.024)	(0.026)	(0.029)
**Information Effect**								
Number of Deaths	0.038**	0.036**	-0.009	0.040**	0.043**	0.025**	0.044**	0.049**
(natural logarithm)	(0.010)	(0.004)	(0.010)	(0.004)	(0.005)	(0.006)	(0.007)	(0.008)
Fear of Infection	0.021	0.001	0.017	-0.001	0.017	-0.031	0.027	-0.000
	(0.038)	(0.012)	(0.022)	(0.014)	(0.015)	(0.019)	(0.024)	(0.027)
Sample Size	2,279	11,900	4,375	9,804	9,644	4,535	4,788	3,083
Number of Individuals	799	3,523	1,282	2,982	3,097	1,583	1,615	975
**(b) Telework**	Age Group		Region		Employee Status		Firm Size	
	20–39	40+	Tokyo plus three neighboring prefectures	Others	Regular Employee	Nonregular Employee	Less than 500	500+ or Public Agencies
	(1)	(2)	(3)	(4)	(5)	(6)	(7)	(8)
**Intervention Effect**								
State of Emergency	0.148**	0.079**	0.127**	0.086**	0.111**	0.075**	0.083**	0.127**
	(0.022)	(0.010)	(0.024)	(0.010)	(0.013)	(0.013)	(0.011)	(0.017)
Quasi-State of	0.011	0.008	0.020	0.024	0.016	0.042*	0.040*	0.019
Emergency	(0.033)	(0.014)	(0.022)	(0.020)	(0.020)	(0.017)	(0.017)	(0.024)
**Information Effect**								
Number of Deaths	-0.043**	-0.006	-0.027*	-0.009	-0.013*	-0.022**	-0.016**	-0.018*
(natural logarithm)	(0.010)	(0.004)	(0.012)	(0.005)	(0.006)	(0.007)	(0.005)	(0.008)
Fear of Infection	0.023	-0.003	-0.028	0.018	-0.019	0.025	0.006	-0.021
	(0.025)	(0.012)	(0.021)	(0.013)	(0.016)	(0.017)	(0.013)	(0.022)
Sample Size	1,838	6,979	2,829	5,944	4,426	2,935	4,410	2,934
Number of Individuals	688	2,313	926	2,014	1,482	996	1,556	953

Note: A 500-employee threshold for firm size was adopted given the survey design.

Robust standard errors in parentheses.

Statistical significance:

**p< 0.01 and

*p< 0.05.

For teleworking, the SOE coefficient was higher among young people, residents of Tokyo and its three neighboring prefectures, regular employees, and those in large firms and government agencies. Although the coefficients for the number of deaths were negative and statistically significant, as mentioned above, the coefficients were extremely small, close to zero.

## Concluding remarks

Based on the household panel data, we confirmed the intervention and information effects on the adoption of preventive behaviors at the individual level, with the information effect being much larger than the intervention effect. This result is in accordance with the literature that claims that individuals change their behaviors for two reasons, a response to the virus threat and a response to government interventions, with the former being more important [2, 3, 25, 26]. We also confirmed that only the declaration of an SOE had an effect on telework implementation. In addition, we found that the SOE had not only a direct intervention effect but also an indirect announcement effect, so that it induced people to more regularly wash their hands.

Although SOEs and quasi-SOEs in Japan were less enforceable and more lenient than those in other countries, they had a certain effect on promoting preventive behaviors and telecommuting during the pandemic. In this sense, government interventions to prevent the spread of infection can be justified. However, even without SOEs and quasi-SOEs, people changed their prevention behaviors based on information about COVID-19 deaths and their fear of infection. This suggests the importance of how and when information should be communicated with people to prevent the spread of infection. We also verified the necessity of effective forms of communication to deliver the appropriate information according to individual attributes.

This study had certain limitations. First, we used self-reported data, so we cannot ignore the possibility of bias that could have reduced the internal validity. Second, the respondent’s own experience of COVID-19 infection was likely to have a substantial impact on the results. However, as such information is not available in the survey data we used, we could not account for this possibility. Furthermore, given sample attrition and the existence of missing values for variables such as income and educational level, the representativeness of the data may have been affected. Nevertheless, as indicated in the demographic characteristics section, the characteristics of the sample still matched the profile of the Japanese population.

## Supporting information

S1 Dataset Data from Fig 2. (XLSX)

S2 Dataset SOEs, quasi-SOEs and COVID-19 deaths. (XLSX)

## References

[1] Goolsbee A, Syverson C. Fear, lockdown, and diversion: Comparing drivers of pandemic economic decline 2020. Journal of Public Economics. 2021; 193(C). doi: 10.1016/j.jpubeco.2020.104311 33262548 PMC7687454

[2] Watanabe T, Yabu T. Japan’s voluntary lockdown. PLoS ONE 16(6): e0252468. doi: 10.1371/journal.pone.0252468 34111163 PMC8191912

[3] Watanabe T, Yabu T. Japan’s voluntary lockdown: further evidence based on age-specific mobile location data. The Japanese Economic Review. 2021; 72 (3): 333–370. doi: 10.1007/s42973-021-00077-9 34177343 PMC8214931

[4] Chang MS, Osier G, Ishii K. Japan Household Panel Survey (JHPS/KHPS) Sampling Weights. PDRC Discussion Paper Series. 2022; DP2022–003. Available from: https://www.pdrc.keio.ac.jp/uploads/DP2022-003_en.pdf.

[5] Nagasu M, Yamamoto I. Impact of socioeconomic- and lifestyle-related risk factors on poor mental health conditions: A nationwide longitudinal 5-wave panel study in Japan. PLoS ONE. 2020; 15(10): e0240240. doi: 10.1371/journal.pone.0240240 33035239 PMC7546460

[6] Duckworth AL, Peterson C, Matthews MD, Kelly DR. Grit: Perseverance and passion for long-term goal. Journal of Personality and Social Psychology. 2007; 9: 1087–1101. 10.1037/0022-3514.92.6.1087 17547490

[7] Maclean H. Commonwealth human biosecurity powers. [citer 18 September 2023]. In: Parliament of Australia [Internet]. Available from: https://www.aph.gov.au/About_Parliament/Parliamentary_departments/Parliamentary_Library/pubs/BriefingBook47p/HumanBiosecurity.

[8] Davis M, Dedon L, Hoffman S, Baker-White A, Engleman D, Sunshine G. Emergency powers and the pandemic: Reflecting on state legislative reforms and the future of public health response. Journal of Emergency Management. 2023; 21(7): 19–35. doi: 10.5055/jem.0772 37154443 PMC10170300

[9] Haddon C, Lilly A, Hogarth R, Marshall J, Nice A. Government emergency powers and coronavirus. 2020 Mar 19 [cited 18 September 2023]. In: Institute for Government [Internet]. Available from: https://www.instituteforgovernment.org.uk/article/explainer/government-emergency-powers-and-coronavirus.

[10] Mathieu E, Ritchie H, Rodés-Guirao L, Appel C, Giattino C, Hasell J, et al. Coronavirus Pandemic (COVID-19). 2020 [cited 18 September 2023]. In: Our World in Data [Internet]. Available from: https://ourworldindata.org/policy-responses-covid.

[11] Cabinet Agency for Infectious Disease Crisis Management. COVID-19 information and resources. 2021 [cited 18 September 2023]. In: Our World in Data [Internet]. Available from: https://corona.go.jp/emergency/pdf/kinkyujitaisochi_20210419.pdf.

[12] The Nihon Keizai Shimbun. What is quasi-State of Emergency? What is the difference between State of Emergency and quasi-State of emergency? 2021 [cited 09 September 2023]. Available from: https://www.nikkei.com/article/DGXZQODL087Q30Y1A400C2000000/.

[13] NHK News Web. What is quasi-State of Emergency? 2021 [cited 09 September 2023]. Available from: https://www3.nhk.or.jp/news/html/20210331/k10012946781000.html.

[14] Ministry of Health, Labour and Welfare. Visualizing the data: information on COVID-19 infections. 2023 [cited 10 September 2023]. Available from: https://covid19.mhlw.go.jp/en/.

[15] Hagger MS, Hamilton K. Social cognition theories and behavior change in COVID-19: A conceptual review. Behaviour Research and Therapy. 2022; 154. doi: 10.1016/j.brat.2022.104095 35605335 PMC9005242

[16] Statistics Bureau of Japan. 2020 Population Census. [cited 09 September 2023] Available from: https://www.e-stat.go.jp/en/stat-search/file-download?statInfId=000032144438&fileKind=0.

[17] Ministry of Health, Labour and Welfare. Overview of the Comprehensive Survey of Living Conditions 2021. 2022 [cited 10 September 2023]. Available from: https://www.mhlw.go.jp/toukei/saikin/hw/k-tyosa/k-tyosa21/index.html.

[18] Statistics Bureau of Japan. 2020 Population Census: Japanese population and households by life stage. 2023 [cited 08 September 2023]. Available from: https://www.stat.go.jp/data/kokusei/2020/kekka/life.html.

[19] Yan Y., Naito T, Tabe Y, Ito K, Nojiri S, Deshpande GA, et al. Increased delta variant SARS-CoV-2 infections in a highly vaccinated medical center in Japan. Vaccine. 2022; 40(23): 3103–3108. doi: 10.1016/j.vaccine.2022.04.029 35465978 PMC9001195

[20] Baek J., Kim K.H. & Choi J.W. Determinants of adherence to personal preventive behaviours based on the health belief model: a cross-sectional study in South Korea during the initial stage of the COVID-19 pandemic. BMC Public Health. 2022; 22(944). doi: 10.1186/s12889-022-13355-x 35546392 PMC9092036

[21] Clark C, Davila A, Regis M, Kraus S. Predictors of COVID-19 voluntary compliance behaviors: An international investigation. Global Transitions. 2020; 2:76–82. doi: 10.1016/j.glt.2020.06.003 32835202 PMC7318969

[22] Uddin S, Imam T, Khushi M, Khan A, Moni MA. How did socio-demographic status and personal attributes influence compliance to COVID-19 preventive behaviours during the early outbreak in Japan? Lessons for pandemic management. Personality and Individual Differences. 2021; 175. doi: 10.1016/j.paid.2021.110692 33526954 PMC7839830

[23] Muto K, Yamamoto I, Nagasu M, Tanaka M, Wada K. Japanese citizens’ behavioral changes and preparedness against COVID-19: An online survey during the early phase of the pandemic. PLoS One. 2020; 15: e0234292. doi: 10.1371/journal.pone.0234292 32525881 PMC7289361

[24] Uchibori M, Ghaznavi C, Murakami M, Eguchi A, Kunishima H, Kaneko S, et al. Preventive Behaviors and Information Sources during COVID-19 Pandemic: A Cross-Sectional Study in Japan. International Journal of Environmental Research and Public Health. 2022; 19(21). doi: 10.3390/ijerph192114511 36361391 PMC9658992

[25] Allen DW. Covid-19 Lockdown Cost/Benefits: A Critical Assessment of the Literature, International Journal of the Economics of Business. 2022; 29(1): 1–32. doi: 10.1080/13571516.2021.1976051

[26] Abouk R, Heydari B. The Immediate Effect of COVID-19 Policies on Social-Distancing Behavior in the United States. Public Health Reports. 2021;136(2):245–252. doi: 10.1177/0033354920976575 33400622 PMC8093844

